# Hardness Assessment Considering Nitrided Layers Based on Tempering Tests for Numerical Wear Prediction for Forging Processes

**DOI:** 10.3390/ma15207105

**Published:** 2022-10-13

**Authors:** Bernd-Arno Behrens, Kai Brunotte, Hendrik Wester, Uwe Lorenz, Felix Müller

**Affiliations:** Institute of Metal Forming and Forming Machines, Leibniz University Hannover, 30823 Garbsen, Germany

**Keywords:** forging, wear calculation, hardness modeling, nitrided layer, Archard model, tool life

## Abstract

The nitriding of forging tools is an industrially established standard used to increase the hardness of the tool surface layer and reduce wear. However, this modification of the tool surface layer, as well as the microstructural changes that occur during this operation due to the thermo-mechanical load, cannot be considered during wear calculations with the widely used Archard wear model in the context of FE simulations. Based on previous work, this study further develops two tempering tests for the investigation of the hardness evolution of two nitride profiles based on H11 tool steel. Here, significant tempering effects could be observed depending on temperature, mechanical stress superposition and time. The results are used for setting up a new material model that is implemented in an existing numerical wear model. The validation is carried out in two laboratory forging test series. The evaluation shows that the hardness development in terms of tempering effects of a nitrided forging tool can be numerically predicted, especially for high forging cycles. However, due to the unexpected occurrence of adhesion effects, only limited applicability of the wear prediction then carried out is achieved.

## 1. Introduction

Long tool life is essential for cost-efficient, safe and resource-saving production. Therefore, in the field of hot forging, a large number of developments aim at increasing the abrasive wear resistance of dies. This involves further developments of tool steels [1] or the development of process measures, such as increasing the semi-finished product temperature up to 1250 °C to reduce the flow resistance and, subsequently, the load on the die [2,3]. Another important approach to enhancing the wear resistance of forging tools focuses on surfaces or surface layer treatments to increase the material hardness significantly. Examples of this approach are carburisations to increase the carbon content in the surface zone, or nitriding treatments in which nitrogen is incorporated in the near surface layer [4]. Nitriding processes in particular are already established in the industrial production of hot-formed components as a standard for increasing the wear resistance of shaping tool components [5,6]. 

During the course of the nitriding process, nitrogen atoms from the surrounding ionised gas atmosphere diffuse into the octahedral gaps of the iron microstructure. The contained iron and the alloy components react by forming nitrides [7]. Of particular importance are chromium–nickel (CrNi) carbides, which lead to a significant increase in surface hardness due to their extreme strength [8]. In general, this type of surface treatment results in a gradual layer system, consisting of a hard compound layer, a diffusion zone and the base material [9]. In the wear-relevant compound layer, it is possible to increase the base hardness from 550 HV0.05 to over 1000 HV0.05 [10]. Using this surface treatment method, in a study of appropriately treated H11 (DIN 1.2343) tool steel, the wear rate was reduced by up to 50% [11]. According to Hawryluk, the expected service life of a nitrided tool in industrial use increases by approx. 10% to 15% [6,12]. Depending on the service life of the tools, tool costs can account for 15–40% of the total process costs and are therefore of great economic relevance [4].

For further reliability and increased efficiency during production planning, the numerical wear calculation is of great interest in process development. With the help of models by Archard and Holm [13,14], which are already implemented in many commercial FE applications, an estimation of tool wear is already possible on the basis of contact variables such as contact pressure, sliding speed and, above all, hardness [15]. However, a problem arises when the described surface layer modification by means of nitriding influences the hardness in such a way that the conventional Archard model, which assumes a constant hardness, is no longer applicable. This is already problematic when the re-hardening and tempering behaviour of the near surface layer, which also occurs in the hot forging process, should be taken into account. 

Within the preliminary work, a user framework was developed and implemented for the FE application Simufact.forming 16.0 (by simufact engineering gmbh, Hamburg, Germany). This enables a numerical prediction of dynamic hardness changes in the near surface layer taking into account the mechanical load described by the equivalent stress according to Mises as well as the temperature at a defined process state (characterised by the number of cycles). Furthermore, this user implementation enables the direct further use of the hardness prediction for a wear and service life calculation [16]. In order to achieve a reliable hardness prediction, a new material characterisation method was developed for this application in previous work [17], with which the hardness evolution of tool steels triggered by the cyclic thermo-mechanical load can be realistically simulated on hollow samples. The validation of this implementation has so far been carried out based on industrial forging dies, which were, however, nitrided in advance for commercial use. Due to this, an interface has already been created in the preliminary work in order to not only reference dynamic hardness curves of the base material, but also to implement a dynamic hardness model for the nitrided layer based on the calculated wear depth [16]. Krawcyk et al. already investigated the thermal softening behaviour of nitrided layers of forging tools using isothermal tempering tests and laboratory forging tests [18]. By varying the temperature between 500 °C and 700 °C for 2 h and 4 h, significant annealing effects (hardness reductions of the nitrided layers) could be observed. In further laboratory forging tests, Krawcyk et al. [18], Widomski et al. [19] and Gronostajski et al. [20] were able to show that the thermo-mechanical load during die forging also leads to annealing effects in the nitrided surface layer. However, since the thermal load on the surface layer is superimposed by abrasive wear and plastic deformation in these tests, a comparison of the two tests (tempering tests and forging tests) is only possible to a limited extent.

**Figure 1:**
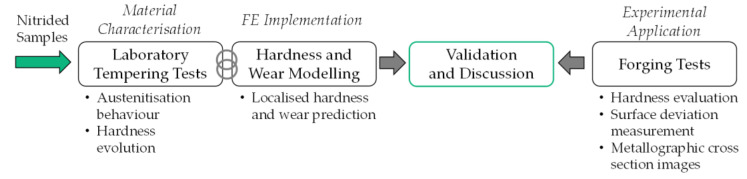
Research plan for the methodology of this study.

When reviewing the cited literature, it is noticeable that studies on the topic of nitrided forging tools either focus strongly on the technological layer development or rather on the possible application advantages of a nitrided layer, e.g., in terms of service life. Specifically, there is a lack of research in the literature that covers the entire process chain from the nitrided layer setup, the recording of transferable material data and the application or implementation of a numerical wear prediction accompanied by a comprehensive experimental forging series for validation purposes. Therefore, following the research plan listed in Figure 1, in this study the experimental characterisation of two nitriding profiles for analogue modelling of the hardness evolution is performed by applying dynamic thermo-mechanical loading based on the method presented in previous work [17]. However, thermo-mechanical cyclic loading proved to have a negative effect on the integrity of the specimens, leading to premature failure of the test. Therefore, isothermal tempering tests with mechanical stress superposition were also carried out. The hardness-time curves determined in these tests were then implemented in an updated implementation of the wear model described above. Analogous to the previous work, the austenitisation behaviour of the nitrided tool steel is also investigated under variation of the stress superposition. This enables the determination of the temperature process limit at which re-hardening effects occur instead of hardening-decreasing tempering effects. Finally, for validation purposes, an experimental application of nitrided and non-nitrided forging tools is carried out enabling a comparison and discussion of the experimental-numerical findings. 

## 2. Materials and Methods

### 2.1. Sample Preparation

The characterisation of nitrided tool surface layers in the context of thermo-mechanical loading firstly requires a preparation of suitable samples. In order to enable a test in the forming simulator DIL 805D, hollow cylinder specimens (outer diameter *D*_a_ = 4 mm, inner diameter *D*_i_ = 3.2 and length *L* = 10 mm) are used. These allow for an experimental simulation of dynamic temperature profiles featuring high heating and cooling rates. Based on previous work, corresponding plates of tool steel H11 (DIN 1.2343) with a thickness of 10 mm were prepared and hardened to a hardness of 450 HV. Subsequently, hollow cylinder specimens with the specified dimensions were taken by means of electric wire erosion. These samples were then further treated using a conventional plasma nitriding process [21]. However, difficulties regarding the homogeneity of the microstructure occurred in this process leading to an iterative process design. In order to achieve a broader focus of the planned experiments, the preparation of two nitriding profiles is proposed. These are to be differentiated by their nitriding intensity, which will be adjusted in the course of this study via the duration of the treatment. It is assumed that a long time of treatment leads to a nitrogen-saturated surface layer which, in contrast to a shorter treatment, features a lower hardness. However, it is assumed that longer treatment times result in a higher robustness against thermo-mechanical loading in terms of hardness reductions due to tempering [10].

**Figure 2:**
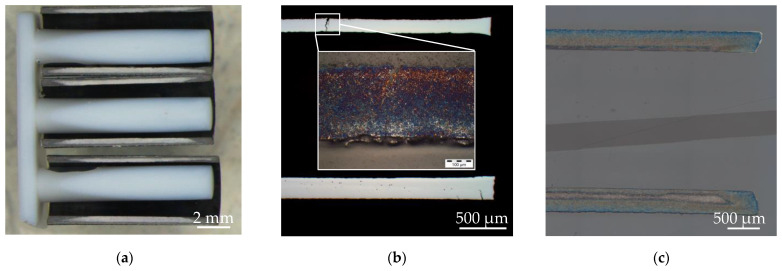
Sample preparation process: Iteration 1 (**a**): Macroscopic sample image showing inadequate nitriding; Iteration 2 (**b**): Stereo and light microscopic image showing cracks after nitriding process; Iteration 3 (**c**): light microscopic image showing a crack-free specimen with near continuous nitriding.

Following the initial sample production process of the hollow cylinder samples, further nitriding was carried out in a plasma nitriding furnace. The samples were arranged and separated in a custom-made grid frame so that the ionised nitrogen could penetrate the surface layer as unhindered as possible. However, the macroscopic image of a metallographically prepared sample length cut from the hollow specimen (Figure 2a) showed a clearly pronounced, inhomogeneous distribution of the nitriding, which was considered problematic for further evaluability of the specimen. In the next step, a further heat treatment step was therefore added to the plasma nitriding process. At a slightly higher temperature for 48 h without further addition of nitrogen, a homogenisation of the nitriding profile was aimed for. In fact, this effect could be achieved, as shown in Figure 2b, but a large part of the samples already showed clearly visible stress cracks, which ruled out their use in further experiments. The relatively thin-walled profiles of the specimen in combination with the extensive heat treatment history of the specimen (hardening process, plasma nitriding and annealing) were identified as the cause of the stress cracks. Nonetheless, in order to generate usable specimens for further investigations, a new iteration was carried out in which the initial case hardening of the specimens was spared. As a result of this iteration, samples with improved nitriding profiles and with no thermal pre-damage could be produced, as shown in Figure 2c.

In the further study, two nitriding profiles were prepared from unhardened H11 tool steel samples, which differ in nitriding time and are referred to below by the designations “32 h PN” and “64 h PN”. The specific treatment parameters of all iterations and variants are summarised in Table 1. 

### 2.2. Test Design for Material Characterisation

**Figure 3:**
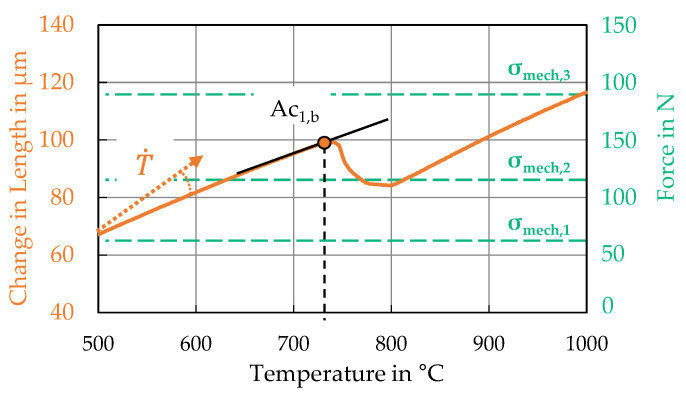
Testing process and evaluation scheme of time-temperature-austenitisation tests.

Firstly, analogous to the investigation of the basic tool steel H11 in [17], continuous heating (time-temperature-austenitisation) tests using a forming dilatometer (DIL 805D by TA Instruments) were carried out. While varying the heating rate $\dot{T}$ and the mechanical stress superposition *σ*_mech_ by applying force, the austenitisation temperature *Ac*_1,b_ is evaluated according to Figure 3. 

While in the previous study the stress component was specified relative to the yield strength of the base material, in this study the applied compressive stress is specified directly in MPa for better transparency. The reason for this is that due to the different yield strengths of all sample variants, a relative representation is no longer appropriate. Thus, the test parameters of the experiments are summarised in Table 2. 

**Figure 4:**
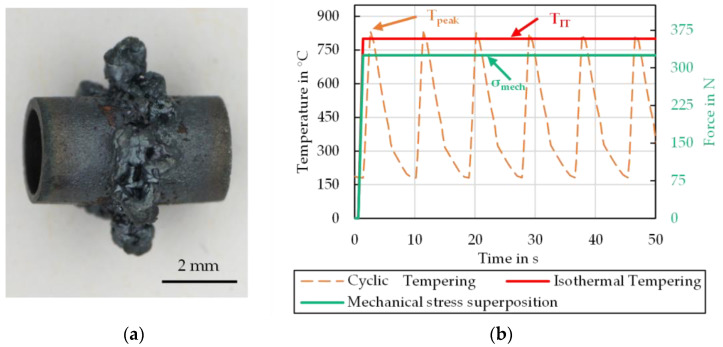
(**a**) Collapsed specimen after 15 cycles at 900 °C peak temperature testing, (**b**) Time-temperature loading profiles for the cyclic and isothermal tempering tests.

In order to obtain suitable measurement data to model the intended dynamic hardness evolution functions, the characterisation method presented in [17] was initially carried out in an analogous manner. This involves the hollow cylinder specimens also being subjected to a cyclic load with a temperature profile (Figure 4b) in the forming dilatometer. In addition to varying the peak cycle temperature (*T*_peak_), a static force can also be applied to the front surfaces of the specimens via the punches of the dilatometer, resulting in the specified compressive stress values *σ*_mech_ when putting the force in relation to the specimen cross-section. In order to ensure a stable test procedure, all tests were carried out at a heating rate ($\dot{T})$ of 250 K/s. The mechanical stress superposition is set analogously to 125 MPa to ensure comparability of the results to the preliminary work [17]. In this study it was also proven that the variation of the stress superposition strength does not lead to a measurable and differentiable result in the cyclic thermo-mechanical tempering tests, so a further variation of the force/pressure influence is therefore not applied. All test parameters are listed in Table 3 and are also marked in Figure 4b.

During the execution of these tests, plastic deformations followed by brittle fractures of the specimens increasingly occurred with higher cycle peak temperature and especially under mechanical stress superposition (Figure 4a). This kind of problem was already observed in the previous work at repetition numbers above 1500. The main reasons were found in unavoidable manufacturing tolerances of the specimen, phase transformation induced plastic strains and the thermal creep effect. Since such a failed specimen could no longer be evaluated, isothermal annealing tests with mechanical stress superimposition were carried out in the dilatometer as part of the presented work. 

These tests exclude the cyclic effects on the microstructural transformation, which is a major cause of specimen failure. Assuming that thermally controlled diffusion is the main mechanism influencing the hardness change of the nitrided specimens, these tests are mainly intended to generate additional results at higher temperatures where existing methods cannot be applied. Under the assumption that both the real forging cycle and the thermal load cycle take less than 10 s, the isothermal tempering time was set to between 10 min and 180 min in order to generate a comparable thermo-mechanical load with the tests in any case (Figure 4b). The investigated temperature range was defined by selecting four discrete stages (*T*_IT_) between 600 °C and 900 °C. As a summary, all test parameters for the isothermal experiments are listed in Table 4 and are also marked in Figure 4b.

**Figure 5:**
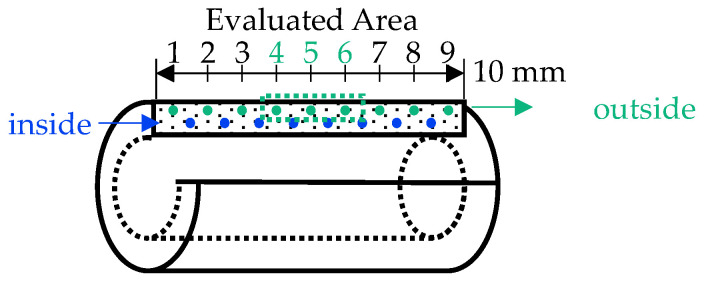
Measuring points for all presented hardness data.

In the context of the study presented here, the evaluation of all tests carried out is primarily based on the resulting hardness at several measuring points in the longitudinal cross-section of the samples. Since the diffused nitrogen displaces the dissolved carbon in the microstructure [22], a carbon-saturated core is inevitably created in each wall of the specimen with the treatment route presented. In this study, carbon (over-)saturated areas are explicitly not considered any further. Since nitrogen accessibility increases via the outer surfaces of the specimen during treatment compared to via the inner surfaces, the core location tends to shift towards the inner side of the specimen wall. For this reason, a series of measurements is taken on each specimen in longitudinal section near the inside and the outside of the wall during the hardness evaluation (Figure 5). For subsequent data modelling, only the measurement results from the outer-center area of the specimen (Points 4, 5 and 6) are considered, in which an approximately homogeneous nitriding profile can be ensured. The results from the inside measuring series are used to monitor the sample quality with regard to the necessary microstructure homogeneity.

### 2.3. Laboratory Forging Tests 

**Figure 6:**
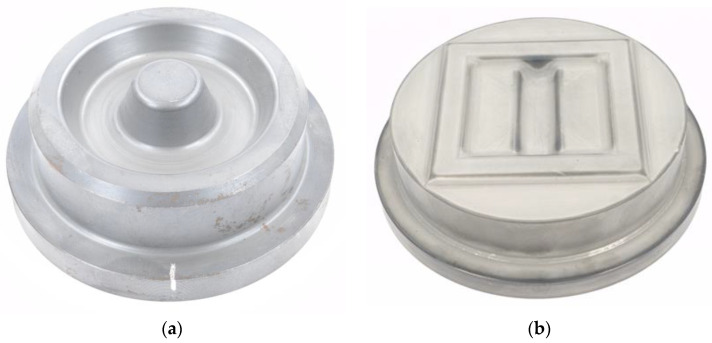
Selected forging die geometries for the laboratory validation processes: (**a**) Type 1 for further validation of the H11 base material data set, (**b**) Type 2 for the validation of the material data of the nitriding profile.

Forging tests planned for the validation of the hardness and wear calculations are carried out under reproducible test conditions in a fully automated forging cell on an eccentric press type SP30d (by SMS Eumuco, Manfort, Germany). As indicated in the introduction, two die geometries are investigated in this study (Figure 6). Die type 1 (Figure 6a) causes high thermal and mechanical loads on the mandrel geometry. This forging series is used to validate the re-hardening and tempering behaviour of the basic tool steel H11 in more detail, before investigating the newly gathered nitriding material data. The dies of type 1 were quenched and tempered to an initial hardness of 45 + 1 HRC. 

As the re-hardening behaviour of the nitriding profiles is not of key relevance—in advance of the results on austenitisation and tempering behaviour presented in Section 3.2—the die geometry of type 2 (Figure 6b) was used for the validation of the numerical hardness and wear prediction. For this die geometry, it is known from numerical preliminary work that surface temperatures of approx. 750 °C are achieved depending on the combination of process parameters. Because of this reduced thermal load compared to die type 1, the risk of thermo-mechanically induced cracking is reduced, which is a typical failure case for nitrided forging tools. For the planned investigations, the dies of type 2 were quenched and tempered to a hardness of 45 + 1 HRC and then treated with the nitriding profile “32 h PN”, as this variant has a higher application relevance due to the shorter treatment time.

For all experiments, the dies were preheated to a starting temperature of 200 °C using heating cartridges. Due to the cyclic heat input of the semi-finished products by the forming process, the dies reach a stationary base temperature of 250 °C after a few cycles as indicated by embedded thermocouples. The billets are made of sawn cylinders using the heat-treatable steel 42CrMo4 (AISI 4140) with the dimensions Ø 30 mm × 40 mm. Prior to forming, they are inductively heated to a temperature of 1150 °C in a push-through induction furnace. The heated billets are automatically transferred into the press chamber by a robot. After each forging cycle, the formed workpiece is ejected and the scale remaining on the tools is removed with compressed air. This is followed by the automatic application of cooling-lubricant by spraying the tool engraving with a water-graphite suspension (Berulit 906 HP from Carl Bechem GmbH, Hagen, Germany, 10% by volume). These processes are repeated cyclically. The cycle time is approx. 5 s. 

To determine the geometric deviations of the tool contours after forging, measurements are conducted using a 3D profilometer (type VR-3200 by Keyence, Osaka, Japan). For this purpose, the surfaces of the forging dies are captured three-dimensionally before and after the tests to enable a pre/after comparison. To determine the geometrical deviation, the after test state is virtually subtracted from the pre-state of the tool surface. Additionally, the tools are cut by wet sectioning and examined metallographically in cross-sections via light microscopy. For further characterisation of the structural changes, hardness depth curves are determined in the near surface zone. These are carried out by Vickers hardness tests.

Finally, Table 5 summarises the test parameters of the laboratory series forging tests. For the basic tests with the non-nitrided tool geometry (Type 1), a forging series was carried out in order to validate relevant cycle numbers from the recorded hardness evolution curves. The basic data show significant changes in hardness at 100 and 500 cycles, while the used die after 2000 cycles is to be investigated primarily with regard to wear characteristics. For the nitrided tool set (Type 2), an additional program stage after 1000 cycles was planned to minimise the risk of a loss of validation data due to unpredictable die failure.

### 2.4. Numerical Process Modelling

**Figure 7:**
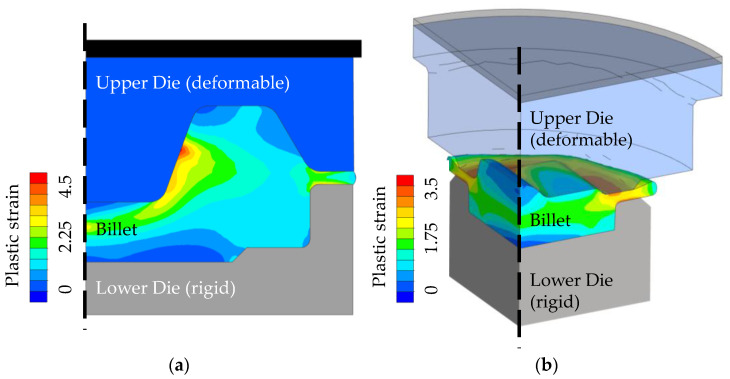
FE-Process model at the end of forming for dies (**a**) Type 1 and (**b**) Type 2.

In order to perform numerical hardness and wear predictions for the described laboratory forging dies, FE process models were necessary, which are depicted in Figure 7. Both models are built up within the FE software Simufact.forming 16.0 using an implicit solver. Taking advantage of the respective symmetry properties, the forming process is modelled as a 2D rotationally-symmetrical model for geometry type 1 and as a 3D ¼ section model for die geometry type 2. 

Following this setup, the 2D model is built up using quad elements while the 3D process is created by employing tetragonal elements. The upper dies are defined as deformable parts in both cases to allow for a more precise calculation of the resulting contact stresses. The lower dies are modelled as rigid tools to reduce the computation time. The attached kinematics of the crank press are derived from the laboratory application. Friction is set for all steps using a constant friction factor of m = 0.4 based on the shear (or tresca) friction model. The starting temperatures of all components are set to 250 °C to map the stationary process state described in Section 2.3. The thermo-mechanical material properties of the 42CrMo4 (AISI 4140) billet are derived from JMatPro. As can be seen in Figure 7, a maximum plastic strain of approx. 4.5 is achieved at the end of forming with die type 1 (Figure 7a), while a maximum plastic strain of 3.5 is set with die type 2 (Figure 7b). The goal of these FE process models is to generate the input data like temperatures and stresses used for hardness and wear calculations. The modelling and implementation of these calculations is noted in the following section.

### 2.5. Wear Modeling and Numerical Implementation

As described in the introduction, this study uses the wear model implemented in the previous work. Based on a modified Archard approach [13,23], this enables an incremental wear calculation to be carried out directly during a process simulation. The equation consists of the wear for the specific time increment $\Delta w_{\mathrm{inc}}$, the wear constant k, the local dynamic hardness change *H*(*t*,*T*), the contact normal stress $\sigma_{N}$ and the sliding velocity $v_{rel}$ in combination with the increment time Δ_*t*_:

(1) $$\Delta w_{\mathrm{inc}}=k\;\cdot \;\frac{\sigma_{N\;}\;\cdot {\;v}_{\mathrm{re}\;l}\;\cdot \;\Delta t}{H(t,T)}$$

However, the chosen implementation strategy in Simufact.forming 16 (based on a UWEARINDEX and UPSTNO user routine) was optimised in this study to improve the calculation time. Based on the fact that the thermal load in hot forming processes steadily rises until the end of the forming process, or at the end of the respective process calculation, the general process calculation strategy is changed to the concept of a decoupled calculation. In this case, the process calculation is first carried out conventionally without any user routines and all relevant calculation results (wear according to Archard, temperature and v. Mises stress at each node) from the last incremental step are transferred to a separate simulation step. This simulation is then configured in such a way that within only three increments a quasi-negligible cooling of 0.1 s is considered. Within these three increments, all calculation laws for hardness and wear determination (published in detail in [16]) are implemented according to the execution order noted in Table 6.

By restricting the total wear implementation to three discrete calculation increments, a significant improvement in the calculation performance is achieved in practice. While the existing implementation for a complete process calculation resulted in a time penalty of approx. 10%, which accumulates to several minutes in each case, the optimised implementation takes less than 30 s using single core calculation. It should also be emphasised that if a change of, e.g., the hardness model for a different material is desired during use, no new time-intensive forming simulation is necessary, but only a recalculation of the decoupled cooling step.

Especially when used in the context of 3D process calculations, there is a further advantage. By implementing the data import in the hardness and wear calculation in the second increment, a smoothing of the temperature field is possible in the first increment. This effect is achieved by exploiting the thermal conduction to eliminate overshooting temperature nodes, which is difficult to prevent in complex process calculations, so that a better comprehensible wear calculation can be achieved.

## 3. Results

### 3.1. Austenitisation Behaviour

**Figure 8:**
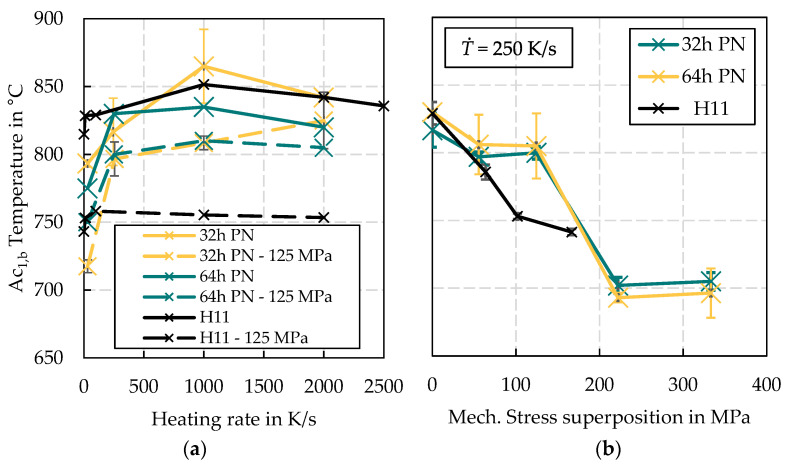
Results of the austenitisation tests: (**a**) Variation of heating rate, (**b**) Variation of mechanical stress superposition.

Following the test plan listed in Table 2, the results of the austenitisation study are shown in Figure 8. For a better overview, the results of the H11 base material published under [17] are also added to both diagrams. By varying the heating rate (Figure 8a), all considered variants show a qualitatively similar austenitisation behaviour, characterised by the evaluated A_C1,b_ temperatures. At a low heating rate of 10 K/s, the earliest austenitisation occurs in all cases. From 100 K/s, a significant step-up can be seen, from which the influence of the heating rate on the A_C1,b_ temperature decreases significantly. Without stress superposition, the 32 h PN variant shows a similar austenitisation behaviour compared to the H11 base material. The 64 h PN variant ranges about 25–50 °C below the results of the 32 h PN variant over the considered heating rate range. It is interesting to note that when a stress superposition is set, not only the A_C1,b_ temperature is reduced but also the influence of the treatment variant almost disappears. This becomes clearer in the results shown on the right (Figure 8b), where the strength of the amount of stress superposition is increased gradually while the heating rate is kept constant.

Considering the standard deviation of approx. 50 °C of the measurement results, it can be concluded in general that the A_C1,b_ temperature decreases nearly linearly with increasing stress superposition. Given the fact that the samples do not represent a completely homogeneous nitrided structure, but also contain non-nitrided, carbon-saturated areas, it can be concluded that this microstructure component essentially determines the austenitisation behaviour in the general picture.

### 3.2. Dynamic and Isothermal Tempering Tests with Mechanical Stress Superposition

Before evaluating the tempering tests, the hardness of the samples after 32 h and 64 h PN treatment described in Table 1 is noted in Table 7. These are the reference values for further tests. It can be seen that the longer treatment period (64 h PN) leads to a reduction in hardness and measuring deviation compared to the shorter treatment (32 h PN).

In the following section, the results of the dynamic-cyclic tempering tests defined in Table 3 and the isothermal test defined in Table 4 are described. All results are presented according to a uniform scheme. In each diagram, the actual hardness measurement result with the associated standard deviation is shown as an isolated data point. Due to partly significant standard deviations in the results, a moving average was calculated from the individual measured values, which are plotted as data lines in the following diagrams. The data line consisting of a solid line represents a measurement series without mechanical stress superposition, whilst the dashed line represents a measurement series with mechanical stress superposition.

**Figure 9:**
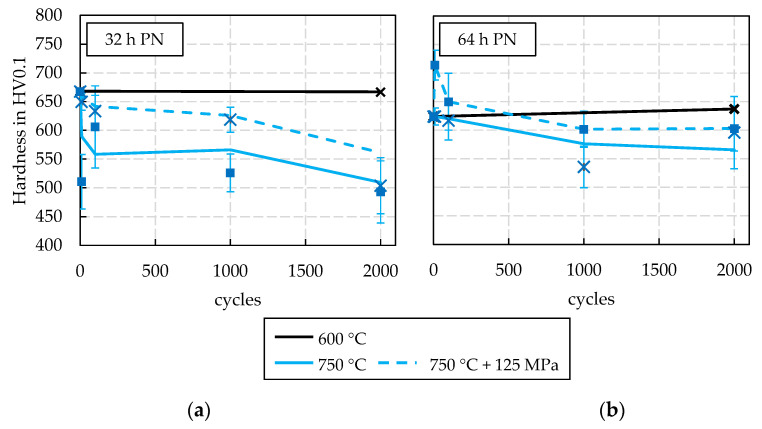
Results of the dynamic, cyclic tempering tests for 600 °C and 750 °C peak temperature: (**a**) 32 h PN, (**b**) 64 h PN.

Considering the results from the cyclic tempering tests with stress superposition shown in Figure 9, it becomes clear that at a peak cycle temperature of 600 °C (black line) no significant change in hardness was observed for either type of PN treatment. This corresponds to the expectations, as the set peak temperature was only 20 °C above the heat treatment during layer preparation (580 °C for 48 h). Based on this, it can be assumed that the temperature increase is too low to trigger further temperature-activated transformation processes in the material, which has been thermally stabilised up to 580 °C. Based on this finding, no further tests were carried out at this peak temperature, as it can be concluded that all areas in the tool surface layer that do not reach 600 °C are not affected by any hardness-relevant change processes. 

The analysis of the tests at a peak temperature of 750 °C (Figure 9) shows a more complex result. In the case of the treatment profile 32 h PN, the stress superposition leads to a clear hindrance of the hardness decrease, as it could already be proven for the base material H11 [17]. For the treatment profile 64 h PN, on the other hand, the stress superposition had no significant influence on the hardness decrease compared to the tests without stress superposition. This result indicates that the microstructure formation is already stabilised after an initial treatment time of 64 h, so that the stress superposition in the subsequent tempering test does not lead to any relevant hindrance of the diffusion processes. Basically, it can be seen that in all tests at a peak temperature of 750 °C, a sudden drop in hardness can be observed after a few cycles, which then changes to a continuous linear hardness decrease. After 2000 cycles, a hardness decrease of approx. 25% and 15% (without vs. with stress superposition) for the 32 h treatment profile, and a hardness decrease of approx. 20% for the 64 h treatment profile, are observed.

**Figure 10:**
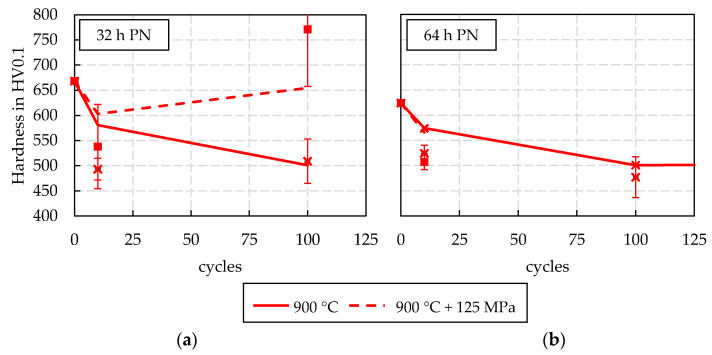
Results of the dynamic, cyclic tempering tests for 900 °C peak temperature: (**a**) 32 h PN, (**b**) 64 h PN.

At a peak temperature of 900 °C, only limited findings are obtained from the cyclic tests (Figure 10). As already described in the methods section, the cyclic thermal load led to a significant, continuous deformation of the specimen (Figure 4a). Due to this, the test could only be carried out stable for 10 to 100 cycles. The evaluation of the test series at 900 °C without stress superposition reveals that no re-hardening effects occur in either nitriding treatment, as there is no noticeable increase in hardness. Therefore, it is concluded that the nitriding structure is tempered in a similar way as in the 750 °C test series.

**Figure 11:**
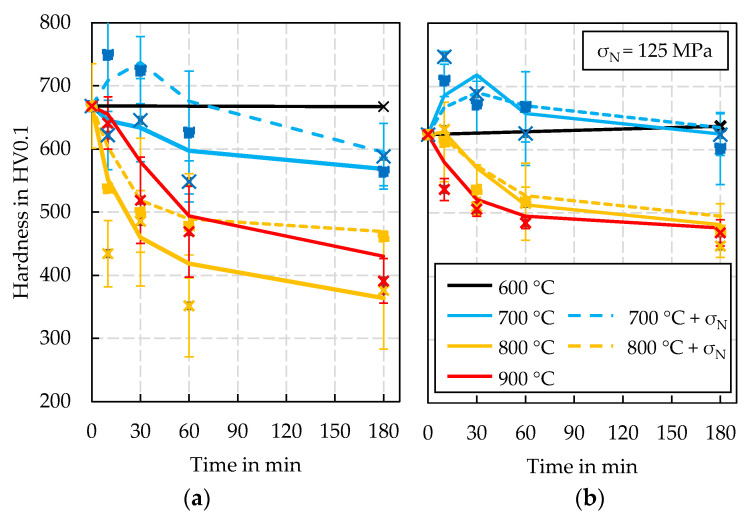
Results of the isothermal tempering tests: (**a**) 32 h PN, (**b**) 64 h PN.

Due to the limitations of the tests described above, an additional testing series was designed and carried out based on an isothermal temperature profile with and without mechanical stress superposition. The aim of this test is to increase the data basis for further hardness modelling. The results of these test series are shown in Figure 11. The diagrams are plotted in the analogous scheme. However, it is noted that the duration of the experiment is displayed in minutes on the x-axis. The results show that the stress superposition has a more significant effect on the samples from the 32 h PN treatment. Compared to the tests without stress superposition, the decrease in hardness due to stress superposition is significantly reduced. The samples from the 64 h PN treatment analogously show that due to the longer initial treatment time, the microstructure has been stabilised to such an extent that the mechanical stress superposition no longer has a significant effect on the course of the hardness. Overall, the isothermal tests show that the duration of the nitriding treatment has a visible influence on the achievable hardness change. For example, at a temperature of 700 °C, a clear hardness reduction of approx. 10% can still be observed after 180 min for the 32 h PN samples. Looking at the 64 h PN samples, this decrease in hardness is not visible after 180 min. On the other hand, it is noticeable that with stress superposition enabled, there is a clear increase in hardness with shorter treatment times, which is then reduced in the course of the tempering process. The test temperature of 800 °C leads to a significant decrease in hardness of up to 40% for both specimen variants (32 h PN, without stress superposition after 180 min).

A significant difference between the two treatment methods can be seen in the results of the tests at 900 °C. For the 32 h PN variant, an abnormal hardness curve was found, which is located between the two data series at 800 °C. This finding is discussed later in depth in Section 4. For the 64 h PN samples, the strongest hardness decrease is recorded for this test temperature. However, the considerable standard deviation of the measurement results must be taken into account here, which amounts to approx. more than ±60 HV0.1 in some cases due to the inhomogeneous microstructure of these specimens. It can therefore be assumed that an increase in temperature above 800 °C no longer leads to a significant increase in hardness-reducing tempering effects and the results can be interpreted as nearly equivalent. Due to a noticeable creep influence, no results with stress superposition could be generated in this isothermal test at 900 °C either, as in all cases there was a direct failure of the sample shortly after the start of the test.

### 3.3. Die Type 1—Validation of Hardness and Wear Prediction for Non-Nitrided H11 Tool Steel

In the following two sections, the hardness prediction is firstly validated in both processes considered. This step is necessary to provide a reliable foundation for the subsequent wear calculation. The evaluation of the hardness prediction is first carried out for die type 1, consisting of the non-nitrided basic tool steel H11, and then for the nitrided die type 2. All predictions are based on the process simulation described in Section 2.5. 

**Figure 12:**
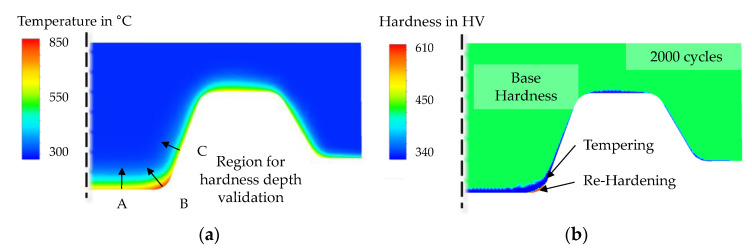
Numerical calculation of (**a**) the temperature field at the end of the forming step and (**b**) a hardness field exemplarily shown for 2000 cycles.

The temperature field of the die type 1 at the end of forming is shown in Figure 12a. Here it becomes clear that a maximum surface temperature of approx. 850 °C is reached under the mentioned process boundary conditions. 

**Figure 13:**
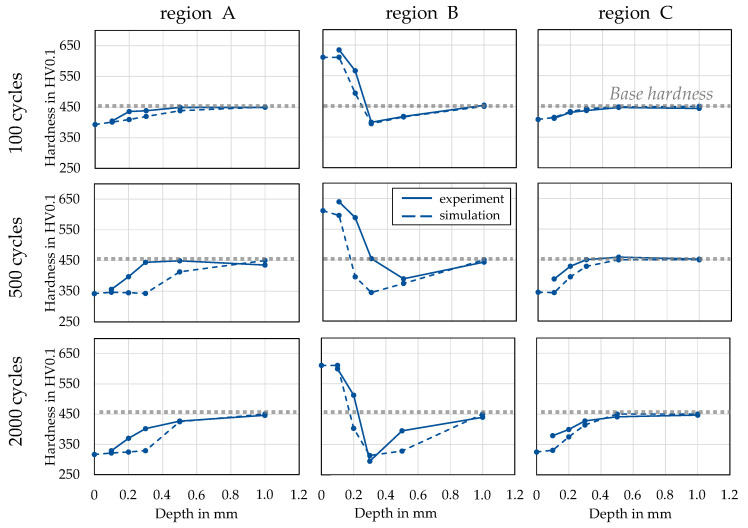
Experimental and numerical results of the hardness evolution after laboratory forging using die type 1.

The further evaluation is focused on the central mandrel area and is carried out by comparing metallographically recorded hardness depth profiles marked as A, B and C (Figure 12a). Figure 13 shows the comparisons of the experimentally determined and numerically calculated hardness depth curves which were obtained directly from the corresponding contour plots, as shown in Figure 12b. Analogous to the test plan, the dies are validated after 100, 500 and 2000 cycles. It can be noted here that for a valid recording of a hardness measuring point, a minimum distance to any edges must be maintained for all cases. For this reason, only the numerically calculated hardness can be specified for the direct surface (0 mm depth). In general, it is clear from the comparison that very good prediction quality was achieved, especially after 100 and 2000 cycles. Notable deviations are only observed after 500 cycles at a depth of 0.3 mm. However, this deviation is negligible for the associated wear calculation, as only the surface hardness is referenced. Deviations of predominantly less than 10% were recorded in all measurement series. 

**Figure 14:**
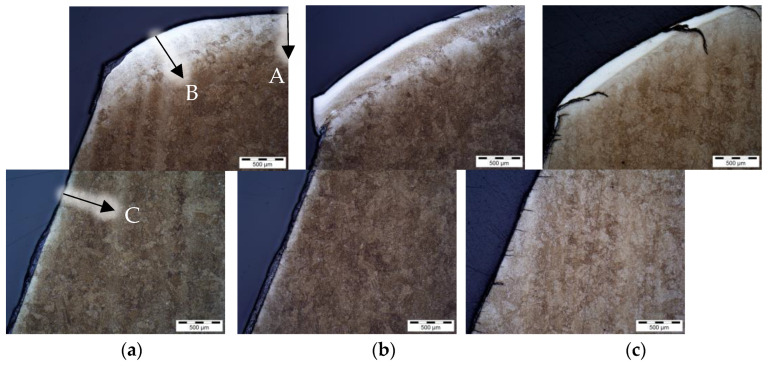
Stitched metallographic images of the tool edge layer of the non-nitrided tool steel H11 after (**a**) 100 cycles, (**b**) 500 cycles and (**c**) 2000 cycles.

Evaluating the hardness predictions shown above, it is evident that tempering effects dominate in the evaluation regions (specified in Figure 14a) A and C, while re-hardening occurs in region B, since the hardness increases at a specific depth. For further validation, etched (Nital 5%) cross-sectional images of the near surface layer after the experiments are shown in Figure 14. 

Due to the etchant used (nitric acid 5%/nital 5%), the re-hardening effects are easily recognisable especially in test region B. Because of the high hardness of the re-hardened zone, a short etching time results in a bright white coloration of the microstructure while the areas with lower hardness appear brown [24]. Underlying tempering effects are recognisable by the tendency towards grain coarsening [25] in comparison to the tempered base microstructure seen in greater depth. In general, the micrographs support the finding on the hardness-depth curves shown in Figure 13.

**Figure 15:**
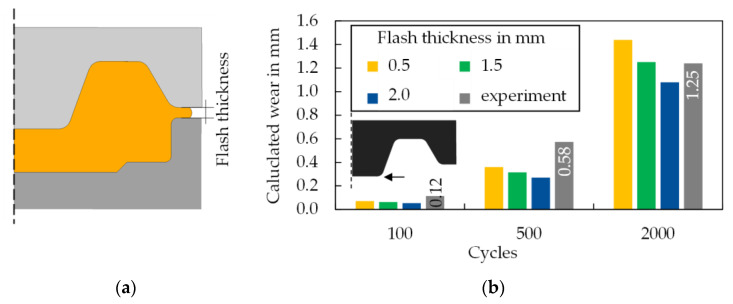
(**a**) Definition of the flash thickness and (**b**) experimental results and numerical wear calculation for die type 1.

Figure 15 shows the results of the numerical and experimental wear evaluation, which was concluded as a subsequent step. Following the optical analysis method described in Section 2.3, the experiments show a continuous increase in material removal (wear) over the number of cycles performed. While the amount of material removed increases linearly by a factor of 5 from 100 to 500 strokes, the wear rate halves up to 2000 strokes, so that after this number of cycles a material removal of 1.25 mm was recorded. 

In numerical wear calculation, it is generally known that the wear or calibration factor *k* has a decisive influence on the quantitative indication of results and that there is no widely accepted and valid procedure for determining this value [26]. For this reason, the calibration factor required for the wear calculation was defined identically in all cases as k = 3 × 10^−7^ according to the literature recommendation already used [27]. Subsequently, a random sample examination of the components produced in the experiment showed that special attention had to be given to the set stroke distance of the die and the associated flash thickness in the process simulation. This parameter is well measurable and indirectly has a considerable influence on the wear calculation, as the load on the tool increases with increasing press stroke and thus decreasing flash thickness with regard to contact pressure, sliding distance and temperature. Following this, the influence of flash thickness (defined in Figure 15a) on resulting wear is illustrated in Figure 15b. Depending on the flash thickness set between 0.5 mm and 2 mm, where 1.5 mm corresponds to the specified thickness, the predicted wear result varies by approx. 10%. It becomes clear that after 100 and 2000 cycles a good prediction of the wear value could be achieved, under the not fully provable assumption that the vast majority of the components had a burr thickness of 1.5 mm. In Figure 14b, after 500 cycles the experimentally determined value deviates significantly from the prediction, which must be attributed to plastic deformations to an undeterminable extent.

### 3.4. Die Type 2—Validation of Hardness and Wear Prediction for 32 h PN Nitrided H11 Tool Steel

Although for the hardness calculation of the non-nitrided tools the material characterisation method could be referenced directly and compared as absolute values, a more complex evaluation is required for the validation of the nitrided tools. 

At first, due to the larger dataset, it was decided that primarily the results from the isothermal tempering experiments would be used for further modelling. However, this leads to the problem that the hardness calculation must be carried out on a discrete cycle basis, whereas the isothermal tempering is continuous time-based. It is therefore necessary to convert the continuous test duration into a discrete number of cycles. For this purpose, it would be conceivable to determine the corresponding tempering parameters according to, e.g., Hollomon–Jaffe [28]. However, according to the literature, this approach has several disadvantages, which are comprehensively summarised in a review by Canale et al. [29]. In this review, it becomes clear that despite the large number of cited studies, the applicability of Hollomon–Jaffe parameters is only given in narrow material groups and, moreover, often only in connection with model adaptations. This is mainly due to the fact that many microstructural effects possess an activation temperature that cannot usually be taken into account by Hollomon–Jaffe approaches. Therefore, in the context of this study, the approach here is to compare the measurement results of the dynamic tempering tests at 750 °C peak temperature with the results of the isothermal tempering at 700 °C and 800 °C. The hardness values obtained after 2000 cycles correspond approximately to the mid-range of the results at 700 °C and 800 °C after 180 min in each case. The global course of the hardness curves is also comparable under this assumption, considering that there is initially a sharp drop in hardness followed by a trend to stationary value. For the hardness modelling of the 32 h PN nitrided layer, the time scales are therefore related in such a way that 180 min in the isothermal tempering correspond to 2000 cycles of the dynamic tempering test.

After the time resolution has been addressed, an adjustment of the hardness representation is necessary. Due to the fact that the quenching and tempering step had to be omitted during specimen production, the nitrided specimens do not achieve the same overall hardness as a conventionally quenched, tempered and subsequently nitrided forging tool. A direct comparison of the hardness would therefore not be conclusive. For this reason, the assumption was made that all microstructural influences in the direct surface layer are dominantly determined by the comparable nitrided microstructure. In the course of the further evaluation, a representation is chosen in which the hardness deviations are related relatively to the respective initial hardnesses. 

**Figure 16:**
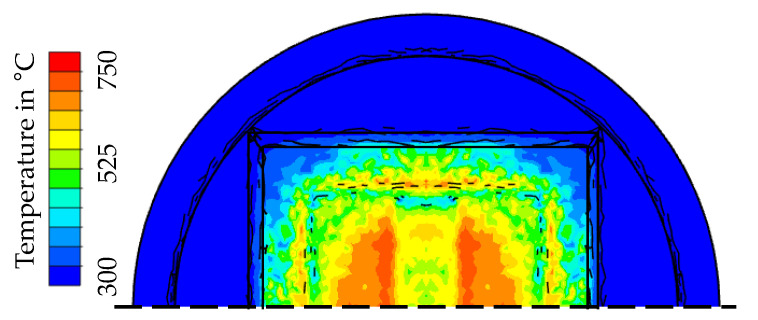
Temperature field of die type 2 at the end of the forming process.

With these assumptions, the further hardness calculation can be carried out analogously to the presentation in Section 3.3 or [16]. Following the approach in Section 3, the surface temperature of the nitrided die is also one of the dominant process variables, which is shown in Figure 16 for the last step of the forming simulation. As expected during the test design, a peak temperature of 750 °C at the central radii is predicted for die type 2, which is significantly lower than for die type 1.

**Figure 17:**
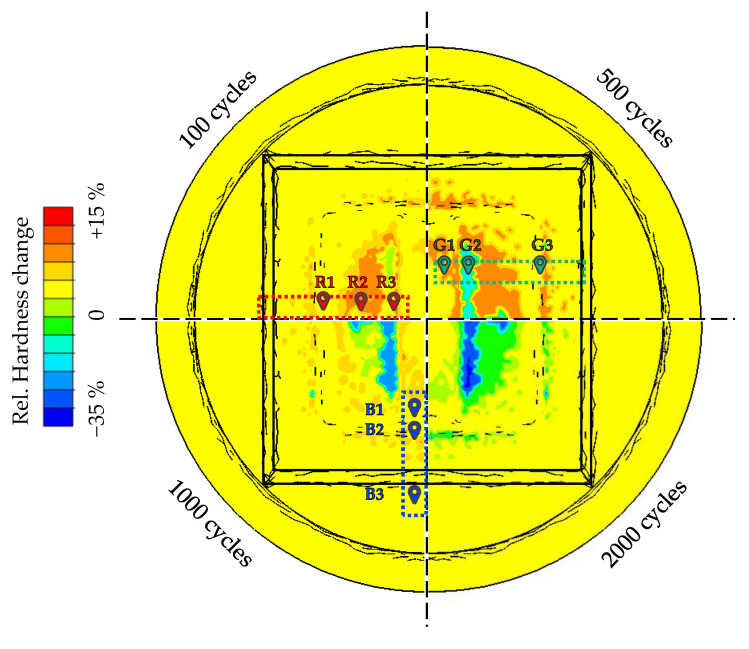
Numerical results of the hardness prediction and overview of the experimental validation points.

Based on the described modelling assumptions, the following figure shows the calculated results of the hardness predictions on die surface for each cycle state considered. To validate this data, the forging dies used were cut by wet sectioning at first and prepared for metallographic examination in cross-sections. Due to tolerances that occur during this process, Figure 17 shows the qualitative areas (coloured boxes) from which the final sections were taken and used for the recording of the required hardness-depth curves. The exact position of the cross-sections was then finally determined by means of previously inserted markers, so that the recorded hardness values for each tool used can be traced back to an exact position in the die, which in turn enables a precise comparison with the calculated simulation results. 

The surface hardness from the forging tools is compared at the marked validation points in Figure 17. Since the validation focus is set on the radii of the die, only these points were selected, for these are of essential relevance for the subsequent wear calculation. A full consideration of hardness depth curves is not validatable due to the previously made assumption that the hardness is only relatively displayable with regard to a reference hardness value determined further below. Therefore, the modelling hypothesis refers exclusively to the wear-relevant near surface area. 

**Figure 18:**
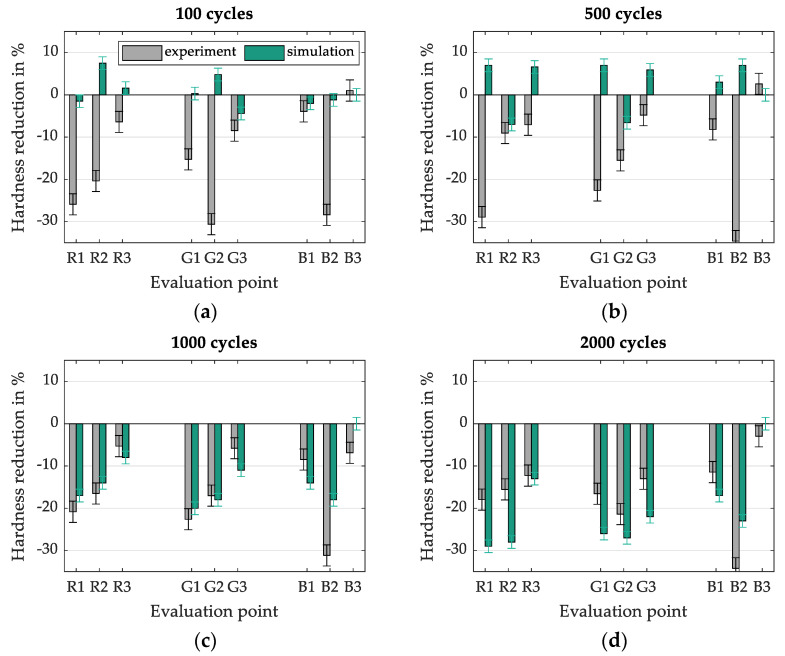
Local evaluation of the measured hardness compared to the numerical results after (**a**) 100 cycles, (**b**) 500 cycles, (**c**) 1000 cycles and (**d**) 2000 cycles.

In the following Figure 18, the respective hardness changes from experiment and simulation are compared for each cycle number and considered evaluation positions. Each result is provided with an error bar, which in this case represents the respective measurement uncertainty of the underlying reference. In the case of the simulation results, this is expressed by the standard deviation of the reference specimens from the material characterisation (668 ± 21 HV0.1). In the case of the forging dies, the reference hardness was determined at several points on the outermost ridge of the die after 100 cycles. This location is characterised by the fact that there is no semi-finished product contact in the process and thus a reference measurement is possible. The reference hardness of the forging dies was determined as 1075 ± 25 HV0.1 using this approach. The evaluation of the local comparison shows that at low cycle numbers (100 and 500 cycles, Figure 18a,b) no recognisable agreement of the experimental and numerical results can be achieved. However, at higher cycle numbers (especially after 1000 cycles, Figure 18c), good qualitative and quantitative agreement is achieved. In contrast to the lower cycle numbers, it is possible to correctly predict tempering effects and, to a large extent, the amount of hardness change in all areas under consideration. 

The reason for this partial prediction validity is most likely due to the fact that the secondary hardness formation seen in the material characterisation tests (Section 3.2) does not occur in the experiments. Instead, the real forging tools directly experience a significant reduction in hardness, which is due to the tempering of the underlying martensitic microstructure. Analogous to the preliminary work on the non-nitrided H11 steel, this effect flattens out after approx. 500 to 1000 cycles and the hardness of the basic microstructure reaches a quasi-stable state. Following this, it can be assumed that the basic microstructure of the material characterisation samples and the forging tools reaches a similar state after 1000 cycles in this study. In conclusion, after this number of cycles, a good prediction accuracy is achieved when comparing the change in hardness.

**Figure 19:**
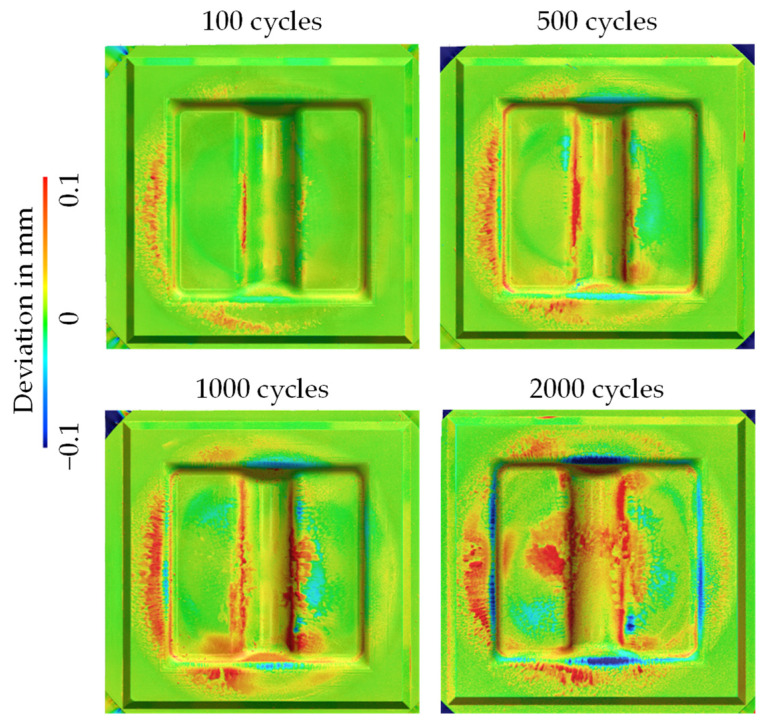
Surface deviation mapping of the die geometry before and after the forging tests.

Following the evaluation of the hardness prediction in Figure 18, Figure 19 shows the results of the geometry comparison of the used forging dies before and after the experiments. Here, it is clear at first sight that, compared to the experiments with die type 1, a significantly reduced material removal is observed. The blue areas show that only approx. 0.1 mm is removed after 2000 cycles. In contrast, it is more noticeable that a significant material adhesion (expressed by positive deviation values) is observable already after 100 cycles, which covers the majority of the die surface after 2000 cycles. 

**Figure 20:**
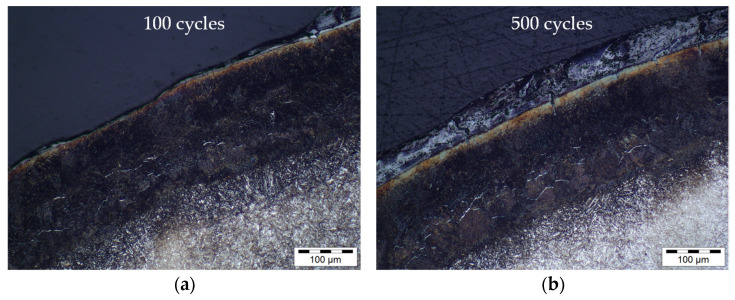

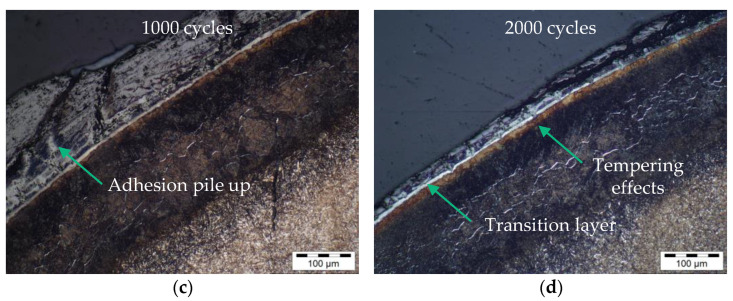
Metallographic cross-section at validation point G2 after (**a**) 100 cycles, (**b**) 500 cycles, (**c**) 1000 cycles and (**d**) 2000 cycles.

With regard to the numerical prediction of wear, material adhesions are problematic, as only material removal can be calculated within the framework of the underlying Archard model. Therefore, the observed adhesion effects cannot be represented and require a different approach. However, when examining the near surface layer (Figure 20, exemplary representation at evaluation point G2), it becomes clear that a tempering microstructure (brown areas) can be found underneath the adhesion layer, which is irregularly strongly formed on each forging die used. This allows the conclusion that the nitriding layer is basically subjected to a tempering behaviour during use, but also that the degree of tempering is related to the adhesion layer. Depending on the layer thickness of these adhesions, it can be assumed that it influences the temperature field in the surface layer, which in turn affects the tempering behaviour. This can also be seen as a reason why the numerical hardness prediction overestimates the real results after 2000 cycles. It is also visible that with increasing numbers of cycles, a white transition layer forms between the adhesion layer and the near surface layer. This shows an influence on the abrasion mechanism which cannot be clarified at this point.

**Figure 21:**
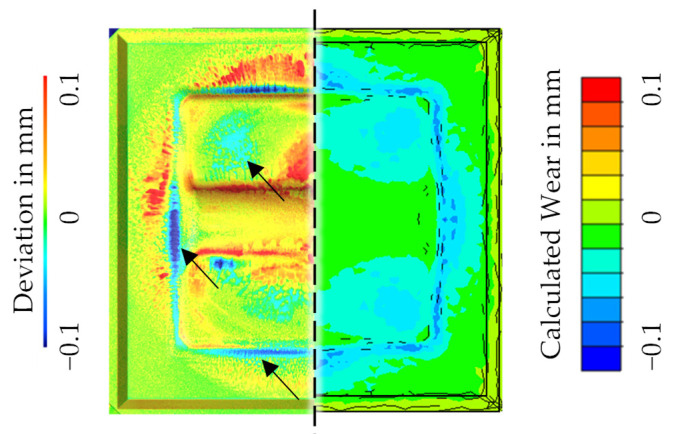
Comparison of the local topography deviations (**left**) and the numerical wear prediction (**right**) for the wear state after 2000 cycles.

Nevertheless, the result of the numerical wear prediction after 2000 cycles is compared with the experimental results in Figure 21. Qualitatively, it becomes clear that at least in the marked areas (black arrow markers) a partial agreement of the wear locations is visible. For the purpose of full disclosure, it is mentioned that the stroke sensitivity of the model for die type 1 also applies here. Therefore, a comprehensive overview of the results is shown for a burr thickness of 1.5 mm, which complies with the statistical average of a sample measurement of the manufactured parts (*n* = 100 parts).

## 4. Discussion

Starting the discussion of the gathered results, an essential challenge in the investigation arises directly at the beginning of the material characterisation with the sample production. The lack of pre-tempering of the samples reduces the heat (pre-)treatment route by a significant step. Observations such as the hardness increases shown in Figure 11 at 700 °C in the course of isothermal tempering can therefore be interpreted as the occurrence of a secondary hardness maximum. Because of the quench and tempering pre-treatment of the nitrided forging die, this effect is not observable in the microstructure after the experiments (Figure 18). 

**Figure 22:**
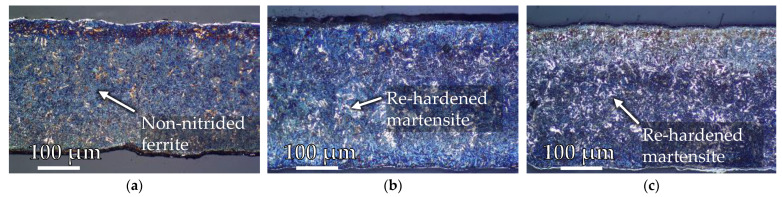
Metallographic sample cross-section (**a**): 32 h PN—reference after nitriding (**b**): 32 h PN, 2000 cycles, 750 °C without stress superposition (**c**): 32 h PN, 2000 cycles, 750 °C with 125 MPa stress superposition.

The investigation on the austenitisation behaviour led to the conclusion that the A_C1,b_ temperature can essentially still be attributed to properties of the base material despite the nitriding and is therefore not directly relevant for the assessment of the nitrided layer properties. This statement is supported by the micrographs in Figure 22. For example, Figure 22a shows the etched (nital 5%) reference microstructure directly after the 32 h PN treatment. Figure 22b,c show the images produced under the same conditions, but from samples after 2000 cycles of dynamic tempering without (b) and with (c) mechanical stress superposition. While the reference (a) shows light brown coloured grains over the entire cross-section, which are interpreted as non-nitrided ferrite with low hardness, the specimens subjected to thermo-mechanical stress at a peak temperature of 750 °C show white grains at similar locations, which can be regarded as re-hardened martensite featuring a high hardness. It becomes clear that with stress superposition, analogous to the results of the austenitisation study (reduction of the *A*_C1,b_ temperature), a significantly increased number of re-hardened martensite grains are visible. The conclusion that the base material primarily determines the austenitisation behaviour would also explain the fact that in Figure 8b no significant difference in *A*_C1,b_ temperature was found between the two nitriding treatment variants.

Since Vickers hardness tests are always carried out on an area of microstructure, it must be assumed that these stochastically distributed ferrite or martensite grains influence the recorded hardness value, which explains the partially significant standard deviations of the measured values after the tempering tests or even generally irregular results as demonstrated by Figure 11. Consequently, the deviations in the numerical hardness prediction, which are mainly limited to the prediction of low cycle numbers, result mainly from the uncertainty of the underlying material characterisation and not from the developed numerical implementation or process simulation. 

In contrast, good agreement between the numerical hardness predictions and the results from the forging experiments was observed when considering the higher cycle numbers. Despite the fact that a direct comparison with other studies is difficult in detail due to partly unknown process boundary conditions and nitrided layer parameters, the observations from the tempering and forging experiments are examined in the following with regard to plausibility on the basis of related studies in the literature. In fact, in analogy to a study by Krawcyk et al. [18], significant reductions in hardness were observed in the nitrided surface microstructure as a result of laboratory isothermal tempering tests. This study also comes to the conclusion that the start of tempering of the nitrided layer investigated can be observed from a minimum temperature of 600 °C. At higher temperatures of 700 °C to 800 °C, on the other hand, hardness reductions (tempering effects) of 30 to 40% were observed after a tempering time of 2 h, which are comparable to the results with purely thermal loading (Figure 11a) in this study. Since tempering tests with mechanical stress superposition are currently unrecorded in the literature, the results of this study can only be compared with the forging tests of Widomski et al. [19], since the real forging applications always feature a thermo-mechanically coupled load. In the context of the study mentioned above, hardness reductions of 20 to 30% were observed in the surface layer of a nitrided forging tool. This range of values agrees very well with the results of the material characterisation (Figure 11a) and the forging tests carried out in this study (Figure 18). The micrographs of the tool edge layer shown by Widomski et al. also feature a qualitatively similar microstructure compared to the micrographs of this paper (Figure 20) with regard to the occurrence of a thin white compound layer and the tempering structure underneath. 

The occurrence of adhesions in the forging experiments presented here was similarly observed by Hawryluk et al. in comparable experiments with nitrided forging tools [30]. Hawryluk et al. argue that the material adhesion of the semi-finished forging product to the tool leads to a significant increase in friction and the associated heat generation. This is one possible reason why, in the context of this study, the highest tempering effects can already be observed after 500 forging cycles (Figure 20b), as a result of the increased thermal load due to the adhesion layer. However, it is also conceivable that, in comparison to the surface states shown in Figure 20, in addition a thinner adhesion layer has formed after 500 cycles, which shields the thermal load from the workpiece to a lesser extent from the actual tool edge layer than in the other views shown. 

Furthermore, an exemplary wear prediction based on the FE-process model for the nitrided tool state after 2000 cycles was demonstrated in Figure 21. Compared with the wear results of the forging process using non-nitrided tools in Figure 15b, it can be concluded, analogous to Widomski et al. [19], that the nitriding of the forging tools resulted in a durability increase (comparison of the wear depths) of approx. 80%. Additionally, when examining Figure 21, it can be observed that in the areas where only slight wear is predicted, especially in the outer area of the flash track, adhesions are observed instead of abrasive wear. This effect is already the subject of recent work of the research team around Frérot et al., who fundamentally examine the interpretation of the Archard wear model in regard to adhesions [31]. Their work features a prediction model for the formation of adhesion based on material and surface parameters and without any other additional fit parameters [32]. However, an application in the context of this study is not directly possible, as a method for recording suitable mechanical strength properties of thin layers needs to be developed first.

## 5. Conclusions and Future Scope

Following very good numerical prediction grades for the hardness and wear calculation based on non-nitrided tool steel H11, the applicability to industrially established nitrided surface layers was investigated in this study, based on the material characterisation method and wear calculation presented in [16,17]. Two nitriding profiles were defined and applied to corresponding hollow cylinder samples. Due to difficulties while conducting the dynamic thermo-mechanically coupled tempering tests, additional isothermal thermo-mechanically coupled tempering tests were successfully carried out. The material data generated in this way were implemented in the numerical calculation framework that was further developed on the basis of the preliminary work and applied in the context of numerical hardness and wear predictions of the forging process. With regard to the hardness prediction, good accuracy could be achieved when higher cycle numbers above 1000 were considered. In this way, it was shown that, in accordance with the literature, a process-relevant thermo-mechanical load can lead to a hardness reduction of 20 to 30% in the surface layer of the forging tool. However, within the numerical wear calculation for the nitrided die (type 2), it was only possible to carry out a partial prognosis due to distinct adhesion effects. Therefore, only a limited validity for the qualitative wear localisation could be achieved.

In the broad context of wear research, and especially numerical wear prediction, the results presented in this study show a significant knowledge gap in the field of experimental process data. The investigations have shown that regardless of the quality of the material characterisation fed into an idealised process simulation, the actual process parameter states (and their variance) during the application determine the actual wear result. Following the stroke influence investigation, it is obvious that similar process variations, such as the billet temperature of the component, which in turn significantly influence the yield stress, lead to a direct influence on the wear. Based on this finding, the authors, together with other scientists of the German Academic Association for Production Technology (WGP), participated in a memorandum for further research on (process) data-based models for the prediction of issues that could not previously be described analytically [33]. In the context of further work, the aim is to generate a broad database of process-relevant data (e.g., various temperatures, press force and timings) for a variety of forging conditions, to be able to map the processes not only on the basis of FE simulations but also on the basis of process-data-based models. In the next step, statistically evaluated process data will be used to specify the loading history of the tool in more detail in terms of the numerical hardness and wear prediction. In the context of this study, it is expected that inconsistencies in the results from the series of forging experiments carried out can be explained in a comprehensible way and taken into account with regard to quantitative predictions. 

## Figures and Tables

**Table 1:** Nitriding process parameters for all sample iterations on H11 tool steel.

Sample Iteration	Base Sample	Plasma Nitriding (PN)	Annealing *
1	case-hardened, 480 HV	550 °C—80% N_2_—32 h	-
2	case-hardened, 480 HV	550 °C—80% N_2_—32 h	560 °C—48 h
3/“32 h PN”	soft-annealed, 280 HV	550 °C—80% N_2_—32 h	560 °C—48 h
3/“64 h PN”	soft-annealed, 280 HV	550 °C—80% N_2_—64 h	560 °C—48 h

* under H_2_—atmosphere.

**Table 2:** Test parameters for the investigation of austenitisation behaviour.

**Name**	**Heating Rate** $\dot{T}$ **in K/s**	**Mech. Stress** **Superposition σ_mech_ in MPa**	**Repetitions**
32 h PN	10, 250, 1000, 2000	0, 66.5, 125, 225, 350	3–5
64 h PN			

**Table 3:** Test parameters for the cyclic thermo-mechanical tempering test.

Name	Peak Temperature *T*_peak_ in ° C	Mech. Stress Superposition *σ*_mech_ in MPa	Cycles	Repetitions
32 h PN	600, 750, 900	0, 125	10, 100, 1000	3
64 h PN				

**Table 4:** Test parameters for the isothermal tempering tests with mechanical stress superposition.

Name	Temperature *T*_IT_ in ° C	Mech. Stress Superposition *σ*_mech_ in MPa	Time in min	Repetitions
32 h PN	600, 700, 800, 900	0, 125	10, 30, 60, 180	3
64 h PN				

**Table 5:** Testing parameters for the laboratory forging tests.

Die Geometry	Base Material	Nitriding Profile	Forging Cycles
Type 1	H11, 45 HRC	-	100, 500, 2000
Type 2	H11, 45 HRC	32 h PN	100, 500, 1000, 2000

**Table 6:** Execution order of the improved wear implementation.

Calculation Increment	Task
1	Initialisation, temperature field smoothing
2	Data import from process simulation into user storage
3	Hardness and wear calculation based on process date at every node

**Table 7:** Hardness of the reference samples after the initial nitriding process.

Specimen	Reference Hardness	Standard Deviation
32 h PN	668 HV0.01	21.4 HV0.01
64 h PN	624 HV0.01	6.2 HV0.01

## Data Availability

The data presented in this study are available on request from the corresponding author. The data are not publicly available due to industrial confidentiality.
